# Rolling-Element Bearing Fault Diagnosis Using Improved LeNet-5 Network

**DOI:** 10.3390/s20061693

**Published:** 2020-03-18

**Authors:** Lanjun Wan, Yiwei Chen, Hongyang Li, Changyun Li

**Affiliations:** 1 School of Computer, Hunan University of Technology, Zhuzhou 412007, China; 2 Hunan Key Laboratory of Intelligent Information Perception and Processing Technology, Hunan University of Technology, Zhuzhou 412007, China

**Keywords:** convolution neural network, LeNet-5 network, fault diagnosis, rolling-element bearing, vibration signals

## Abstract

To address the problems of low recognition accuracy, slow convergence speed and weak generalization ability of traditional LeNet-5 network used in rolling-element bearing fault diagnosis, a rolling-element bearing fault diagnosis method using improved 2D LeNet-5 network is put forward. The following improvements to the traditional LeNet-5 network are made: the convolution and pooling layers are reasonably designed and the size and number of convolution kernels are carefully adjusted to improve fault classification capability; the batch normalization (BN) is adopted after each convolution layer to improve convergence speed; the dropout operation is performed after each full-connection layer except the last layer to enhance generalization ability. To further improve the efficiency and effectiveness of fault diagnosis, on the basis of improved 2D LeNet-5 network, an end-to-end rolling-element bearing fault diagnosis method based on the improved 1D LeNet-5 network is proposed, which can directly perform 1D convolution and pooling operations on raw vibration signals without any preprocessing. The results show that the improved 2D LeNet-5 network and improved 1D LeNet-5 network achieve a significant performance improvement than traditional LeNet-5 network, the improved 1D LeNet-5 network provides a higher fault diagnosis accuracy with a less training time in most cases, and the improved 2D LeNet-5 network performs better than improved 1D LeNet-5 network under small training samples and strong noise environment.

## 1. Introduction

Rolling-element bearing is the key component of mechanical equipment, and the bad and complex working environments can easily cause rolling-element bearing fault during runtime [1]. To ensure the long-term and stable operation of rolling-element bearing, many researches have been done on rolling-element bearing fault diagnosis. The traditional rolling-element bearing fault diagnosis mainly adopts signal processing and machine learning techniques. The vibration signal processing techniques used in rolling-element bearing fault diagnosis mainly include time-domain analysis [2], frequency-domain analysis [3] and time-frequency analysis [4,5,6,7]. The wavelet analysis [4], short-time Fourier transform (STFT) [5], empirical mode decomposition [6] and singular value decomposition [7] are commonly used methods in time-frequency analysis of vibration signals of rolling-element bearing. The machine learning method used in rolling-element bearing fault diagnosis firstly extracts fault features from vibration signals, and then maps the extracted fault features into the fault type of rolling-element bearing. The common machine learning methods for rolling-element bearing fault diagnosis include support vector machine (SVM) [8], k-nearest neighbor (k-NN) [9], K-Means clustering [10], back propagation neural network (BPNN) [11], etc. The traditional rolling-element bearing fault diagnosis methods have been widely used, but with the increasing complexity of vibration signals, these methods have a certain limitation; however, the deep learning methods have a greater advantage in analyzing complicated and non-stationary vibration signals.

The deep learning methods can automatically extract fault features from vibration signals [12], recently there are many researches are conducted on rolling-element bearing fault diagnosis using deep learning. Yin et al. [13] extracted the original features of vibration signals through time-domain analysis, frequency-domain analysis and wavelet transform, and obtained the low-dimensional features from 38 original features using the nonlinear global algorithm, and the low-dimensional features array is input into the deep belief network (DBN) to evaluate the performance status of rolling-element bearing. Liu et al. [14] obtained the spectrogram of vibration signals through STFT, used the stacked sparse auto-encoder (SAE) to automatically extract fault features, and employed the softmax regression to identify the fault type of rolling-element bearing. Liu et al. [15] used the recurrent neural network (RNN) to classify the faults of rolling-element bearing, and adopted the gated recurrent unit based denoising auto-encoder to enhance fault classification accuracy. Among different deep learning methods, compared with DBN, SAE and RNN, the convolution neural network (CNN) has the characteristics of local perception, weight-sharing and subsampling, which can achieve higher performance at a lower cost.

Recently, 2D CNN has been widely used in rolling-element bearing fault diagnosis [16,17,18,19,20,21,22,23,24,25]. Janssens et al. [16] proposed a feature learning method based on 2D CNN for detecting fault of rolling-element bearing, and the accuracy increases by about 6% compared with the random forest classifier. Hoang et al. [17] proposed a bearing fault diagnosis method based on 2D CNN without manual feature extraction, which converts 1D vibration signals into 2D gray images and takes them as input data of the CNN classifier. Lu et al. [18] built a rolling-element bearing fault diagnosis model using a hierarchical 2D CNN, the experiments prove that it can provide higher classification accuracy than using SAE and SVM. Guo et al. [19] investigated a hierarchical adaptive 2D CNN on bearing fault diagnosis, which can automatically and sensitively extract fault features from vibration signals. Fuan et al. [20] proposed an adaptive deep 2D CNN for rolling-element bearing fault diagnosis, and the key parameters of the CNN classifier are determined by particle swarm optimization algorithm. Li et al. [21] proposed a bearing fault diagnosis method based on deep 2D CNN and D-S evidence theory, the results show that it can adapt to different load conditions. Liu et al. [22] proposed a bearing fault diagnosis method using a lightweight 2D CNN, and improved the diagnosis accuracy and generalization ability by adding a BN layer and L2-regularization. Wen et al. [23,24,25] conducted a series of studies on rolling-element bearing fault diagnosis using the state-of-the-art 2D CNN models including AlexNet, VGG-19 and ResNet-50, and the experiments show that they work well in the bearing fault diagnosis field. The existing researches indicate that the fault diagnosis methods based on 2D CNN can get high diagnosis accuracy, but some problems exist such as time-consuming preprocessing stage, high computational complexity, long training time and poor real-time performance.

Compared with 2D CNN, 1D CNN has a simpler network structure and a lower computational complexity, and it directly takes 1D raw vibration signals as input without any preprocessing, so it can provide a faster processing speed and is suitable for real-time fault diagnosis. Recently, there have been many works on rolling-element bearing fault diagnosis using 1D CNN [26,27,28,29,30]. Eren et al. [26,27] developed a bearing fault diagnosis system using the compact adaptive 1D CNN classifier, which directly takes raw vibration signals as input and provides a competitive classification performance. Abdeljaber et al. [28] studied a compact 1D CNN to identify, quantify, and localize ball bearing damage. Zhang et al. [29] proposed a method based on deep 1D CNN to address bearing fault diagnosis problem, it takes raw vibration signals as input and does not need any denoising preprocessing, and the results show that the method performs well in noisy environment and achieves a high fault diagnosis accuracy under different working load. Ma et al. [30] proposed a lightweight deep 1D CNN for rotating machinery fault diagnosis, which has a high training speed and a strong transfer-learning ability.

The LeNet-5 network developed by LeCun et al. [31] is a classic 2D CNN model, which has been successfully applied to Alzheimer’s disease recognition [32], traffic sign recognition [33], facial expression recognition [34], gas recognition [35], pedestrian detection [36] and other fields. Due to LeNet-5 network has a relatively simple structure and a powerful classification capability, this paper employs LeNet-5 network for rolling-element bearing fault diagnosis. Aiming at the problems of low recognition accuracy, slow convergence speed and weak generalization ability in rolling-element bearing fault diagnosis based on traditional LeNet-5 network, this paper proposes a novel rolling-element bearing fault diagnosis method using improved 2D LeNet-5 network, which can provide a rolling-element bearing fault diagnosis model with high classification accuracy, fast convergence speed and strong generalization ability. On the basis of improved 2D LeNet-5 network, this paper proposes an improved 1D LeNet-5 network for rolling-element bearing fault diagnosis, which can greatly reduce the training time and provide better diagnosis accuracy in most cases. The effectiveness of the proposed methods are evaluated through the rolling-element bearing data [37] from Case Western Reserve University (CWRU). The main contributions of this paper are as follows:

- The histogram equalization is carried out on the gray images during the preprocessing of experimental data, which can provide better input data for an improved 2D LeNet-5 network.
- The convolution and pooling layers are reasonably designed and the size and number of convolution kernels are carefully adjusted, which can enhance the fault classification capability of improved 2D LeNet-5 network.
- The batch normalization is used to normalize the output of each convolution layer, and the dropout operation is introduced after each full-connection layer except the last layer, which can improve the convergence speed and generalization ability of improved 2D LeNet-5 network.
- On the basis of improved 2D LeNet-5 network, a well-designed 1D LeNet-5 network is proposed for performing the 1D convolution and pooling operations on the 1D raw vibration signals, which can provide a higher fault diagnosis accuracy with a less training time in most cases.

The rest of the paper is organized as follows. The basic theory is introduced in Section 2. The proposed rolling-element bearing fault diagnosis method using improved 2D LeNet-5 network is described in Section 3. The proposed rolling-element bearing fault diagnosis method using improved 1D LeNet-5 network is discussed in Section 4. The experimental results and analysis are presented in Section 5. The conclusions and future work are given in Section 6.

## 2. Basic Theory

### 2.1. Convolution Neural Network

The convolution neural network is a multi-layer neural network and its unique weight-sharing structure considerably reduces the complexity of neural network, which is generally composed of the input layer, convolution layer, pooling layer, full-connection layer and output layer [38].

The convolution layer consists of several convolution kernels, and the input data is convolved with the convolution kernels to extract features. Assuming that the *l*-th layer is a convolution layer, the convolution process can be described as:

(1) $$x_{j}^{l}=f\left(\sum_{i\in M_{j}^{l-1}}x_{i}^{l-1}*k_{ij}^{l}+b_{j}^{l}\right)$$

where * represents the convolution operator, $x_{j}^{l}$ denotes the *j*-th feature map of the *l*-th layer, $x_{i}^{l-1}$ denotes the *i*-th feature map of the (*l*−1)-th layer, $M_{j}^{l-1}$ is the number of feature maps of the (*l*-1)-th layer which are connected with the *j*-th feature map of the *l*-th layer, $k_{ij}^{l}$ represents the convolution kernel corresponding to the *i*-th feature map of the (*l*−1)-th layer and the *j*-th feature map of the *l*-th layer, and $b_{j}^{l}$ denotes the bias value of the *j*-th feature map of the *l*-th layer.

In order to gradually reduce the parameters of the neural network and prevent over-fitting, a pooling layer is usually added between two consecutive convolution layers, and the robustness of feature extraction is enhanced by using the pooling operator. Assuming that the (*l*−1)-th layer is a convolution layer and the *l*-th layer is a pooling layer, the pooling process can be described as:

(2) $$x_{j}^{l}=f\left(w_{j}^{l}\mathrm{lower}\left(x_{j}^{l-1}\right)+b_{j}^{l}\right)$$

where $w_{j}^{l}$ represents the weights of the *j*-th feature map of the *l*-th layer and lower() denotes the pooling function.

After the input data is processed through several convolution and pooling layers, a full-connection layer combines all the local features of the processed data into the global features for subsequent classification.

### 2.2. Traditional LeNet-5 Network

The traditional LeNet-5 network [31] is originally designed for handwritten digit recognition, which consists of two convolution layers, two pooling layers and three full-connection layers. The detailed settings of traditional LeNet-5 network structure are listed in Table 1.

As illustrated in Table 1, the input data is black-and-white images of 32×32 pixels. The Conv1 layer uses six convolution kernels of size 5×5 to generate six feature maps of 28×28 pixels. The Pool1 layer performs a 2×2 max-pooling operation on the output of Conv1 layer to generate six feature maps of 14×14 pixels. The Conv2 layer uses 16 convolution kernels of size 5×5 to generate 16 feature maps of 10×10 pixels. The Pool2 layer performs a 2×2 max-pooling operation on the output of Conv2 layer to generate 16 feature maps of 5×5 pixels. The FC1 layer is a full-connection layer with 120 neurons, which is fully connected with Pool2 layer and produces 120 feature maps of 1×1 pixels. The FC2 layer is a full-connection layer with 84 neurons, which calculates the dot-product between the input vector and weight vector and adds the bias value, and the results are output by the sigmoid function. The FC3 layer is also called the output layer, which has 10 neurons and divides all the input images into 10 different categories corresponding to numbers 0–9.

## 3. Rolling-Element Bearing Fault Diagnosis Method Using Improved 2D LeNet-5 Network

### 3.1. Process of Rolling-Element Bearing Fault Diagnosis Based on Improved 2D LeNet-5 Network

**Figure 1:**
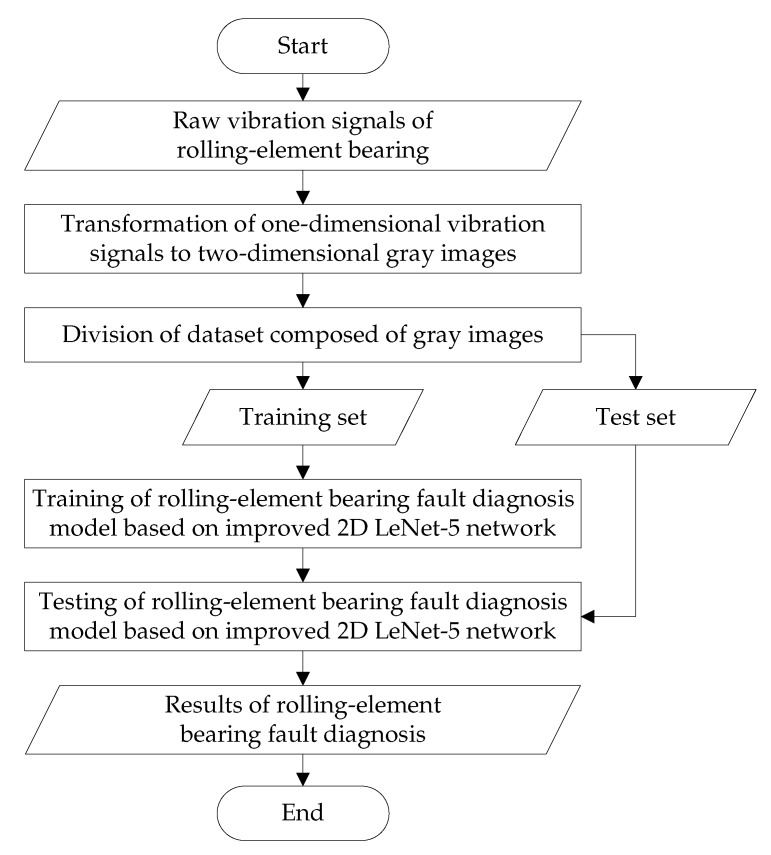
Flowchart of rolling-element bearing fault diagnosis based on the improved 2D LeNet-5 network.

The process of rolling-element bearing fault diagnosis based on the improved 2D LeNet-5 network is shown in Figure 1, which can be described as follows:

Step 1: The vibration signals are collected by sensors deployed on the rolling-element bearing.
Step 2: The 1D raw vibration signals are transformed into the 2D gray images, and the histogram equalization is carried out on the gray images for enhancement.
Step 3: The dataset composed of gray images is divided into the training set and test set.
Step 4: The training set is input into the improved 2D LeNet-5 network for training, and the fault diagnosis model based on the improved 2D LeNet-5 network is obtained.
Step 5: The test set is input into the fault diagnosis model for testing, and the results of rolling-element bearing fault diagnosis are analyzed to evaluate the validity of the model.

### 3.2. Preprocessing of Experimental Data Used in Improved 2D LeNet-5 Network

The experimental data is provided by CWRU [37], and the data used in this paper is collected under 12K and 48K sampling frequencies and motor load of 0, 1, 2, 3 horsepower (HP). Specifically, the experimental data includes the normal condition data, inner-race fault data, ball fault data and outer-race fault data.

The preprocessing of experimental data used in improved 2D LeNet-5 network is similar to the transformation process of signals described in [39], at first every 4096 pieces of continuous raw vibration signals are divided into a sample, and then each sample is divided into 64 equal parts, which are aligned as the rows of the 2D image. In this way, the 1D raw vibration signals with a length of 4096 is transformed into a 2D image with a size of 64×64, and each sample is normalized according to Equation (3) and transformed into a gray image of 64×64 pixels using MATLAB.

(3) $$p_{i}^{\prime }=\frac{p_{i}-p_{\min}}{p_{\max}-p_{\min}}\times 255$$

In Equation (3), $p_{i}$ represents the *i*-th sampling point of the current sample, and $p_{\min}$ and $p_{\max}$ represent the minimum and maximum values of all sampling points of the current sample respectively.

To solve the problem that the local features of gray images are not obvious, the histogram equalization method is adopted to make the distribution of pixel gray values become more uniform and enhance the contrast of images, which is helpful to promote convergence speed and fault classification accuracy of improved 2D LeNet-5 network. The process of performing histogram equalization on a gray image is as follows.

Step 1: The number of pixels of each gray level is calculated according to the gray value of each pixel of a gray image, and the histogram is obtained according to the gray level. The *x*-axis and *y*-axis of histogram represent the gray level and the number of pixels, respectively.
Step 2: All the gray levels whose number of pixels are more than zero are found.
Step 3: The gray level with the least number of pixels is found and denoted as minCDF.
Step 4: The cumulative distribution function (CDF) of each gray level is calculated.
Step 5: The gray value of each pixel which belongs to the gray level whose number of pixels is more than zero is updated by Equation (4), where *M* and *N* represent the length and width of the gray image respectively.

(4) $$p=\frac{CDF-minCDF}{M\times N-minCDF}$$

**Figure 2:**
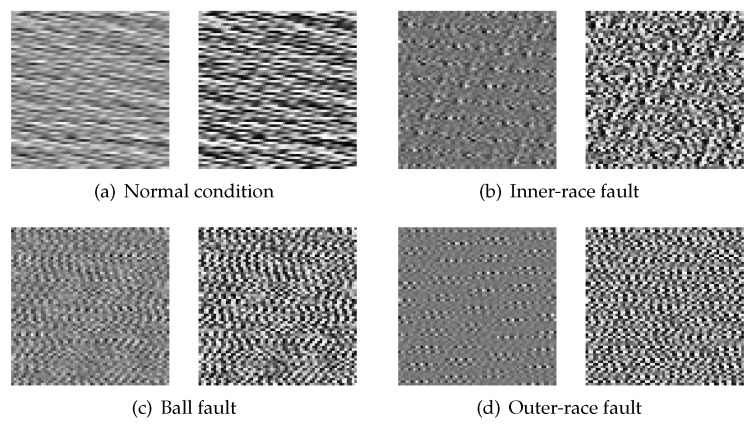
Four gray images with different conditions of rolling-element bearing.

To illustrate the effect of histogram equalization, four different samples under motor load of 1 HP are selected, including one sample with normal condition, one sample with inner-race fault, one sample with ball fault and one sample with outer-race fault. These four samples are transformed into four gray images, as shown in Figure 2. The left side of each sub-figure is the gray image without histogram equalization, and the right side of each sub-figure is the gray image with histogram equalization. Obviously, the histogram equalization method can effectively enhance the contrast of images.

The dataset composed of gray images with different conditions of rolling-element bearing under different motor loads are divided into training sets and test sets according to the ratio of 7:3, as shown in Table 2. The gray images are marked according to different conditions of rolling-element bearing, the normal condition is marked as N, the inner-race fault with fault diameter of 0.007, 0.014 and 0.021 inches are marked as I007, I014 and I021 respectively, the ball fault with fault diameter of 0.007, 0.014 and 0.021 inches are marked as B007, B014 and B021 respectively, and the outer-race fault with fault diameter of 0.007, 0.014 and 0.021 inches are marked as O007, O014 and O021 respectively.

### 3.3. Structure of Improved 2D LeNet-5 Network for Fault Diagnosis

It is observed that the traditional LeNet-5 network used in rolling-element bearing fault diagnosis has low fault classification accuracy, slow convergence speed and weak generalization ability, therefore the following improvements of traditional LeNet-5 network are made.

1. The gray images of 64×64 pixels are used in the input layer. In the training of rolling-element bearing fault diagnosis model, it is found that the smaller the image, the lower the fault diagnosis accuracy, and the larger the image, the slower the training speed. The fault diagnosis accuracy and training speed are comprehensively considered, it is necessary to determine a suitable image size, and the image of 64×64 pixels is selected.
2. One convolution layer and one pooling layer are added. Theoretically, the deeper the neural network, the stronger the feature expression ability, but the more difficult the optimization problem. Three convolution layers and three pooling layers are used in the improved 2D LeNet-5 network, which can extract much more fault feature information and obtain better training effect.
3. The size and number of convolution kernels are changed. The number of convolution kernels of each convolution layer of traditional LeNet-5 network is less, in view of the non-stationarity and complexity of vibration signals, it is necessary to carefully adjust the size and number of convolution kernels to enhance the fault classification capability. The first convolution layer uses eight convolution kernels of size 8×8, the second convolution layer uses 32 convolution kernels of size 8×8, and the third convolution layer uses 64 convolution kernels of size 5×5.
4. The batch normalization is adopted. BN can speed up the convergence, simplify the parameter adjustment and avoid the gradient vanishing problem.
5. The dropout operation is introduced. The dropout operation can effectively prevent and reduce over-fitting during the training of the fault diagnosis model, and improve the generalization ability of the model.
6. The ReLU activation function is used. When computing the error gradient by back propagation, the ReLU activation function can effectively alleviate the gradient disappearance, and it has faster computation speed compared with the sigmoid activation function used in traditional LeNet-5 network, so it can accelerate the training of neural network.

**Figure 3:**
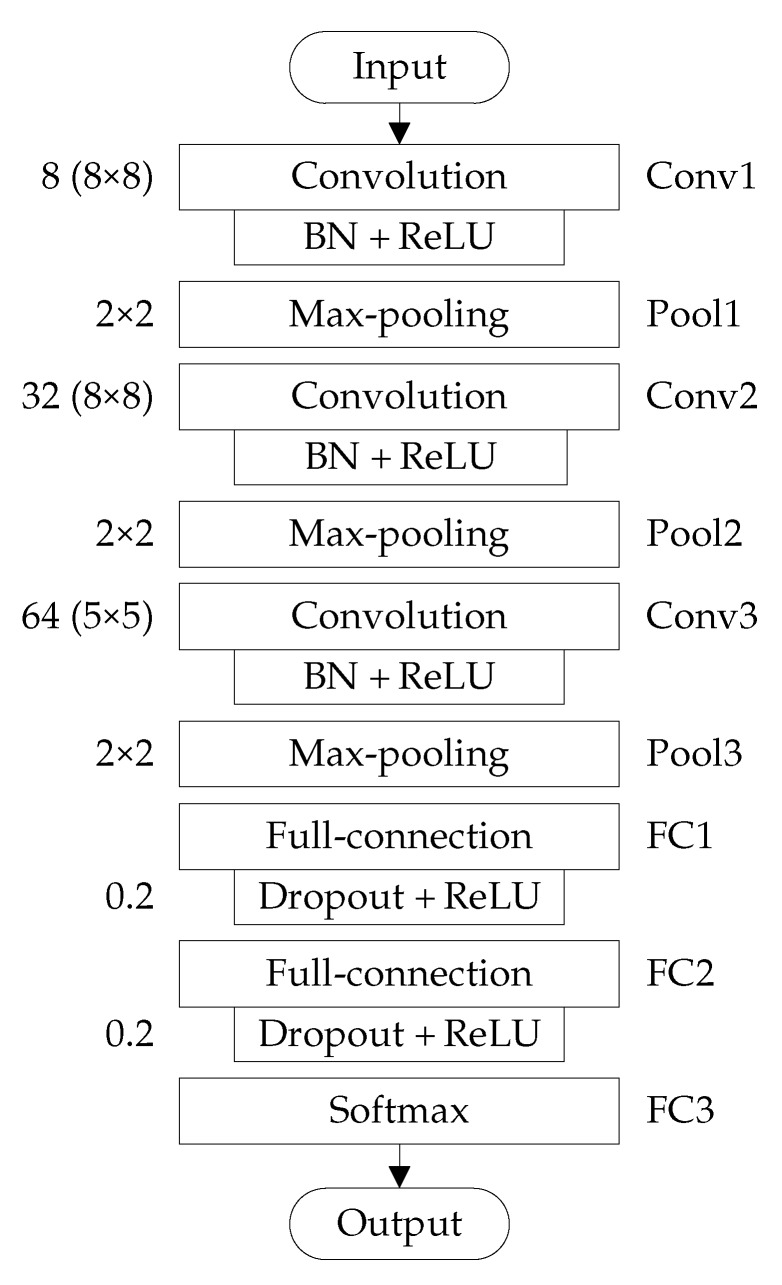
Structure of improved 2D LeNet-5 network for rolling-element bearing fault diagnosis.

The improved 2D LeNet-5 network for rolling-element bearing fault diagnosis has nine layers, as shown in Figure 3, which includes three convolution layers (i.e., Conv1, Conv2 and Conv3), three pooling layers (i.e., Pool1, Pool2 and Pool3) and three full-connection layers (i.e., FC1, FC2 and FC3).

The Conv1 layer performs the convolution operation on the neighborhood of size 8×8 of a gray image of 64×64 pixels with 8 convolution kernels of size 8×8, and 8 feature maps of 57×57 pixels are generated. The Pool1 layer performs the 2×2 max-pooling operation on the neighborhood of size 2×2 of each feature map outputted by Conv1 layer, and eight feature maps of 28×28 pixels are generated. In this paper, the strides of each convolution operation and each pooling operation are set to 1 and 2 respectively, and the padding modes of all the convolution and pooling layers are set to ‘VALID’.

The Conv2 layer performs the convolution operation on the neighborhood of size 8×8 of each feature map outputted by Pool1 layer with 32 convolution kernels of size 8×8, and 32 feature maps of 21×21 pixels are generated. The Pool2 layer performs the 2×2 max-pooling operation on the neighborhood of size 2×2 of each feature map outputted by Conv2 layer, and 32 feature maps of 10×10 pixels are generated.

The Conv3 layer performs the convolution operation on the neighborhood of size 5×5 of each feature map outputted by Pool2 layer with 64 convolution kernels of size 5×5, and 64 feature maps of 6×6 pixels are generated. The Pool3 layer performs the 2×2 max-pooling operation on the neighborhood of size 2×2 of each feature map outputted by Conv3 layer, and 64 feature maps of 3×3 pixels are generated.

The FC1 layer is fully connected with the output of Pool3 layer through 120 neurons, which combines all the local features of feature maps outputted by Pool3 layer into the global features, and 120 feature maps of 1×1 pixels are produced. The FC2 layer is fully connected with the output of FC1 layer through 84 neurons. The FC3 layer (i.e., the output layer) is fully connected with the output of FC2 layer through four neurons, which uses the softmax function to classify the input data into four different categories corresponding to the normal condition, inner-race fault, ball fault and outer-race fault of rolling-element bearing.

After each convolution layer, the BN is adopted to normalize each feature map generated from the convolution operation, which can reduce internal covariate shift and promote the training efficiency of improved 2D LeNet-5 network.

After each of the first two full-connection layers, the dropout operation is introduced and the dropout ratio is set to 0.2, namely the neurons will be temporarily discarded from the neural network with a probability of 20%, which can to some extent restrain over-fitting.

After each convolution layer and each of the first two full-connection layers, the ReLU activation function is used to change all the negative values of each feature map into zero, which can completely backward-propagate the calculated gradient without causing the gradient disappearance.

The detailed settings of improved 2D LeNet-5 network structure for rolling-element bearing fault diagnosis are listed in Table 3.

## 4. Rolling-Element Bearing Fault Diagnosis Method Using Improved 1D LeNet-5 Network

Although the proposed rolling-element bearing fault diagnosis method based on the improved 2D LeNet-5 network has high diagnosis accuracy, fast convergence speed and strong generalization ability, it has the following disadvantages: (i) the transformation of 1D raw vibration signals into 2D gray images is time-consuming; (ii) the multi-layer 2D convolution and pooling operations result in a relative long training time. In order to further improve the efficiency and effectiveness of fault diagnosis, on the basis of improved 2D LeNet-5 network, an end-to-end rolling-element bearing fault diagnosis method based on the improved 1D LeNet-5 network is discussed in this section.

### 4.1. Process of Rolling-Element Bearing Fault Diagnosis Based on Improved 1D LeNet-5 Network

Similar to the process of rolling-element bearing fault diagnosis based on the improved 2D LeNet-5 network, the process of fault diagnosis based on the improved 1D LeNet-5 network can be described as follows: firstly, the vibration signals are collected by sensors deployed on the rolling-element bearing; secondly, the dataset composed of 1D raw vibration signals is divided into training set and test set; thirdly, the training set is input into the improved 1D LeNet-5 network for training, and the rolling-element bearing fault diagnosis model is obtained; finally, the test set is input into the rolling-element bearing fault diagnosis model for testing, and the testing results are analyzed to evaluate the performance of the model.

For the improved 1D LeNet-5 network, the experimental data is also provided by CWRU, and every 4096 pieces of vibration data are divided into a sample. The dataset composed of 1D raw vibration signals with different conditions of rolling-element bearing under different motor loads are divided into training sets and test sets according to the ratio of 7:3, as shown in Table 4.

### 4.2. Structure of Improved 1D LeNet-5 Network for Fault Diagnosis

**Figure 4:**
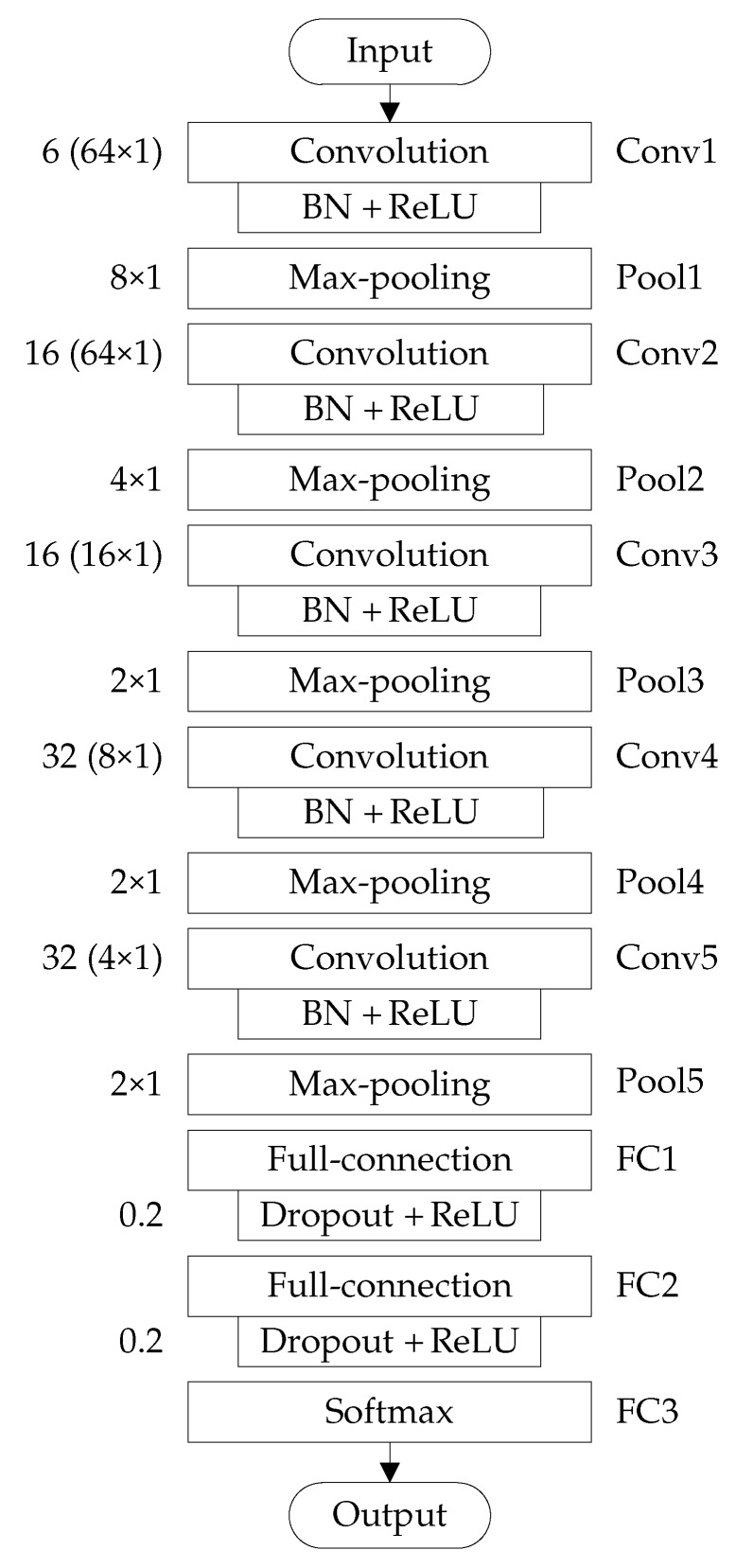
Structure of improved 1D LeNet-5 network for rolling-element bearing fault diagnosis.

The improved 1D LeNet-5 network used in rolling-element bearing fault diagnosis has the similar structure with improved 2D LeNet-5 network, as shown in Figure 4, which includes five convolution layers, five pooling layers and three full-connection layers. The detailed settings of improved 1D LeNet-5 network structure for rolling-element bearing fault diagnosis are listed in Table 5.

Each convolution layer adopts an appropriate number of convolution kernels with suitable size to perform the 1D convolution operation with a stride of one. Specifically, the Conv1 layer adopts six convolution kernels of size 64×1, the Conv2 layer adopts 16 convolution kernels of size 64×1, the Conv3 layer adopts 16 convolution kernels of size 16×1, the Conv4 layer adopts 32 convolution kernels of size 8×1, and the Conv5 layer adopts 32 convolution kernels of size 4×1. Each pooling layer adopts a suitable size of pooling kernel to perform the 1D pooling operation. Specifically, the Pool1 layer performs the 8×1 max-pooling operation with a stride of eight, the Pool2 layer performs the 4×1 max-pooling operation with a stride of four, and the Pool3, Pool4 and Pool5 layers perform the 2×1 max-pooling operation with a stride of one.

After each convolution layer, the BN and ReLU activation function are adopted. After FC1 and FC2 layers, the dropout operation are performed, and the dropout ratio is set to 0.2. The samples composed of 1D raw vibration signals are used in the input layer, and four different conditions of rolling-element bearing (i.e., normal condition, inner-race fault, ball fault and outer-race fault) are recognized by the FC3 layer with four neurons.

## 5. Experimental Results and Analysis

### 5.1. Experimental Setup

The improved 2D LeNet-5 network and improved 1D LeNet-5 network are implemented in MATLAB 2018 and Pytorch 1.1.0 and are tested on a computer with a hexa-core Intel i7-8750H CPU at 2.2 GHz and 16 GB RAM. The parameters settings of improved 2D LeNet-5 network and improved 1D LeNet-5 network are listed in Table 6. All experiments are conducted using stochastic gradient descent (SGD) with momentum, where the initial learning rate is set to 0.008, momentum is set to 0.9 and step-size is set to 0.5 by comprehensively considering convergence speed and classification accuracy. It is important to select suitable batch size during the training and test phases of CNN, so both training batch size and test batch size are set to 128.

### 5.2. Training and Verification of Fault Diagnosis Models

**Figure 5:**
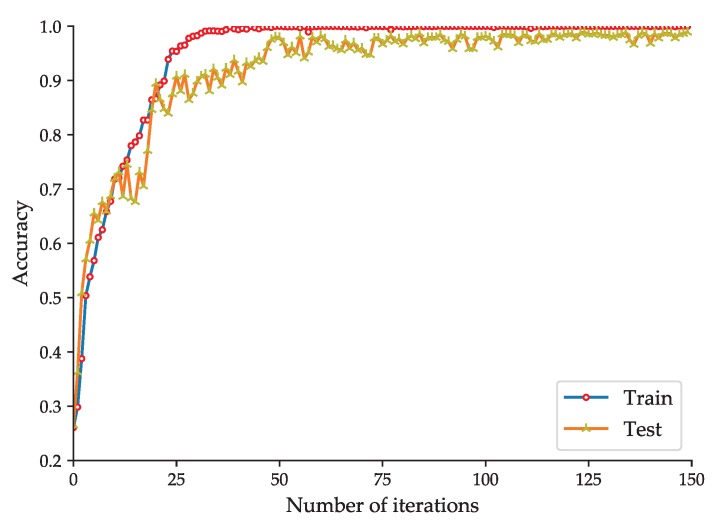
Accuracy curve of the fault diagnosis model based on the improved 2D LeNet-5 network.

**Figure 6:**
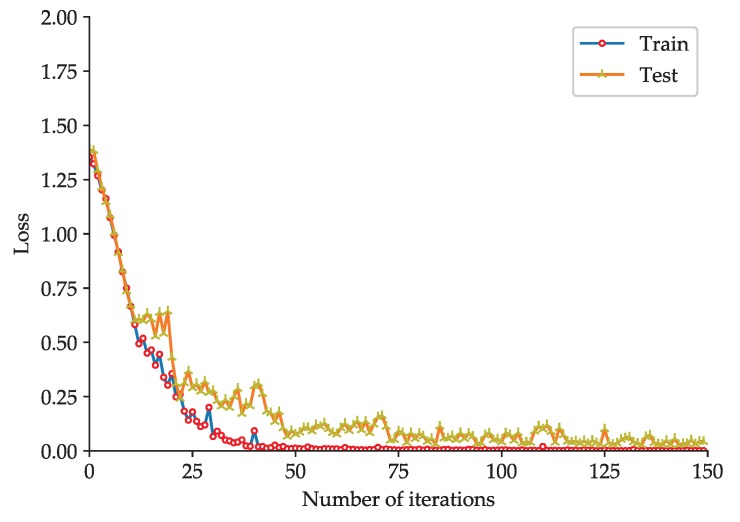
Loss function curve of the fault diagnosis model based on the improved 2D LeNet-5 network.

**Figure 7:**
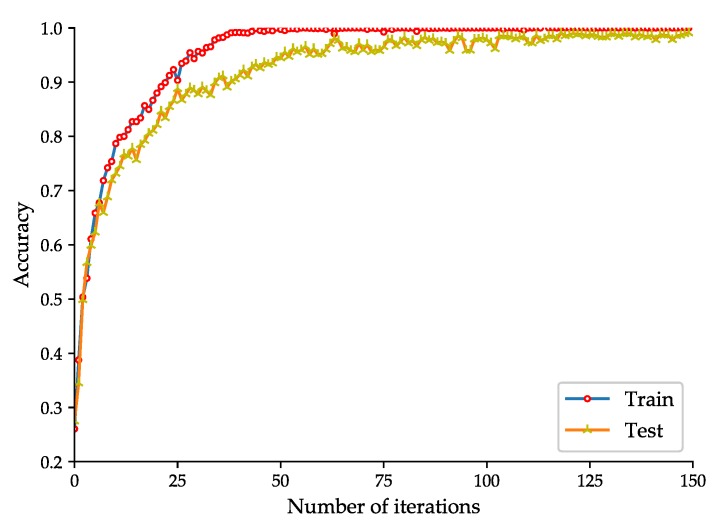
Accuracy curve of the fault diagnosis model based on the improved 1D LeNet-5 network.

**Figure 8:**
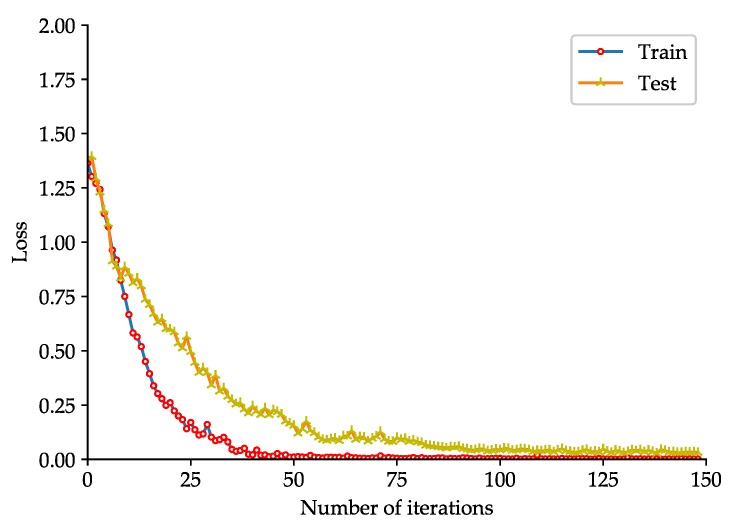
Loss function curve of the fault diagnosis model based on the improved 1D LeNet-5 network.

To evaluate the effectiveness of the proposed rolling-element bearing fault diagnosis models, the fault diagnosis model based on the improved 2D LeNet-5 network is trained and tested with the dataset listed in Table 2, and the fault diagnosis model based on the improved 1D LeNet-5 network is trained and tested with the dataset listed in Table 4. To better observe the training effect of the model, the test of the model is performed at each iteration during the training phase. The accuracy curve and loss function curve obtained during the training and test phases of the fault diagnosis model based on the improved 2D LeNet-5 network are shown in Figure 5 and Figure 6 respectively. The accuracy curve and loss function curve obtained during the training and test phases of the fault diagnosis model based on the improved 1D LeNet-5 network are shown in Figure 7 and Figure 8 respectively.

As shown in Figure 5, during the training and test phases of the fault diagnosis model based on the improved 2D LeNet-5 network, the fault classification accuracy tends to be stable after about 75 iterations. When the number of iterations reaches 150, the accuracy obtained in model test can reach up to 98.66%. If the number of iterations is increased, the accuracy will be slightly improved. As shown in Figure 6, the loss function value decreases rapidly in the first 75 iterations, and then it decreases slowly and closes to zero. It is easy to see that the results presented in Figure 7 and Figure 8 are similar to that presented in Figure 5 and Figure 6. During the test phase of the fault diagnosis model based on the improved 1D LeNet-5 network, when the number of iterations reaches 150, the accuracy can reach up to 99.11%. As can be seen from Figure 5, Figure 6, Figure 7 and Figure 8, the model training results are closed to the model test results in general, and there is no under-fitting or over-fitting. The results prove the effectiveness of the proposed fault diagnosis models.

Table 7 presents the diagnosis accuracy of different conditions of rolling-element bearing. As seen in Table 7, for the improved 2D LeNet-5 network, the average diagnosis accuracy of the normal condition, inner-race fault, ball fault and outer-race fault can reach to 99.73%, 99.19%, 99.10% and 99.31% respectively; for the improved 1D LeNet-5 network, the average diagnosis accuracy of the normal condition, inner-race fault, ball fault and outer-race fault can reach to 99.78%, 99.66%, 99.55% and 99.73% respectively. The overall average diagnosis accuracy of improved 2D LeNet-5 network and improved 1D LeNet-5 network can reach to 99.25% and 99.66%, respectively. The results show that both improved 2D LeNet-5 network and improved 1D LeNet-5 network can effectively classify the normal condition, inner-race fault, ball fault and outer-race fault of rolling-element bearing.

**Figure 9:**
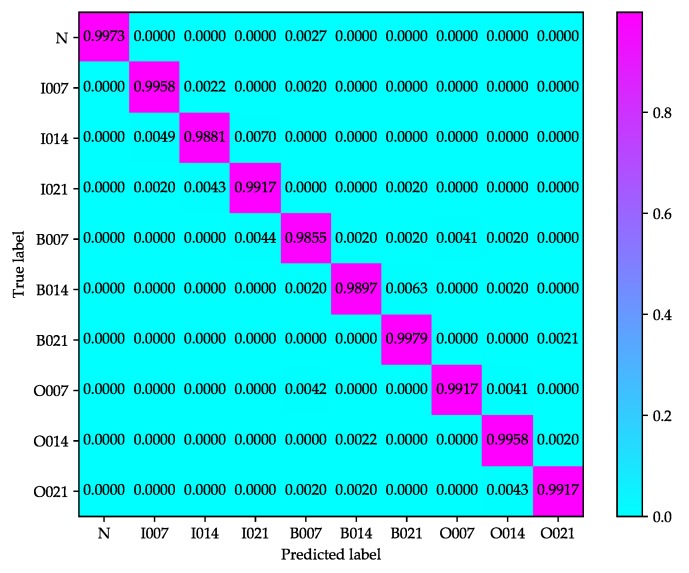
Confusion matrix of test samples used in the improved 2D LeNet-5 network.

**Figure 10:**
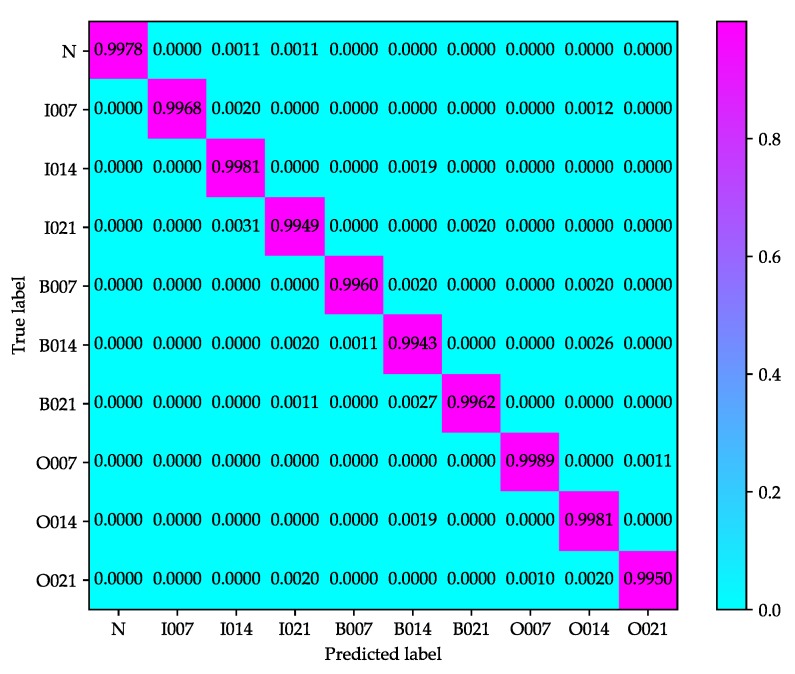
Confusion matrix of test samples used in the improved 1D LeNet-5 network.

Figure 9 and Figure 10 show the confusion matrices of test samples used in improved 2D LeNet-5 network and improved 1D LeNet-5 network respectively, which give the classification results in detail. The rows and columns of confusion matrix stand for the actual label and predicted label of each condition, respectively. As shown in Figure 9, the accuracy of conditions N, I007, I021, B021, O007, O014 and O021 reaches over 99%, but the accuracy of conditions I014, B007 and B014 is relatively lower. B007 receives the most misclassification, where 0.27% out of N, 0.20% out of I007, 0.20% out of B014, 0.42% out of O007 and 0.20% out of O021 are misclassified to B007. As shown in Figure 10, the accuracy of each condition reaches over 99%, but the diagnosis accuracy of ball fault is slightly lower than that of inner-race fault and outer-race fault. B014 receives the most misclassification, where 0.19% out of I014, 0.20% out of B007, 0.27% out of B021 and 0.19% out of O014 are misclassified to B014. It can be noted from Figure 9 and Figure 10 that the improved 2D LeNet-5 network and improved 1D LeNet-5 network not only have high classification accuracy but also have stable classification performance.

### 5.3. Analysis of Impact of BN on Improved 2D LeNet-5 Network

**Figure 11:**
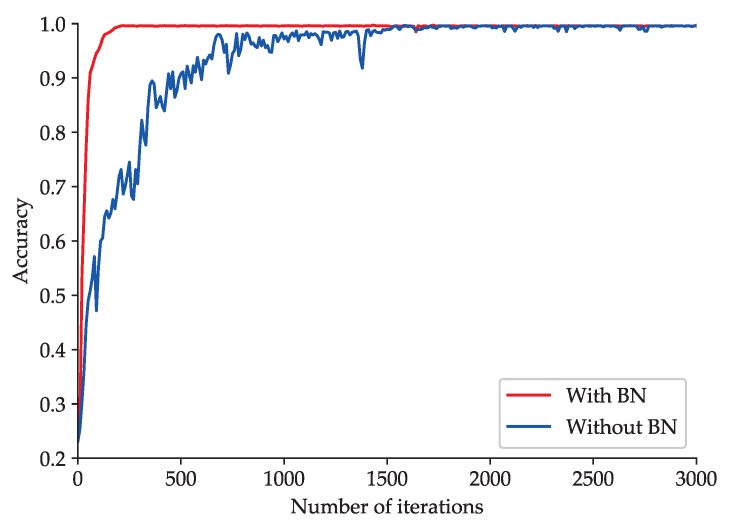
Accuracy curve obtained during the training processes of the improved 2D LeNet-5 network with BN and that without BN.

**Figure 12:**
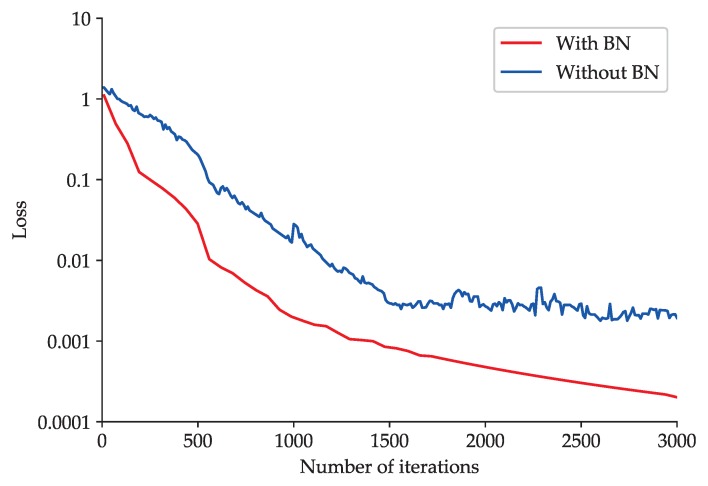
Loss function curve obtained during the training processes of the improved 2D LeNet-5 network with BN and that without BN.

To explore the impact of BN on the improved 2D LeNet-5 network, an experimental comparison between improved 2D LeNet-5 network with BN and that without BN is made. The accuracy curve and loss function curve obtained during the training processes of improved 2D LeNet-5 network with BN and that without BN are shown in Figure 11 and Figure 12, respectively.

As shown in Figure 11, the improved 2D LeNet-5 network with BN can achieve high and stable fault classification accuracy after about 100 iterations. However, the accuracy obtained by improved 2D LeNet-5 network without BN rises slowly and fluctuates greatly, and the accuracy tends to be stable after about 1500 iterations. As shown in Figure 12, when BN is adopted in improved 2D LeNet-5 network, the loss function value decreases steadily and approaches to zero gradually with the increase of the number of iterations; when BN is not adopted, the loss function value decreases relatively slowly. The loss function value of improved 2D LeNet-5 network with BN is roughly an order of magnitude lower than that of improved 2D LeNet-5 network without BN after about 2000 iterations. The results show that BN can significantly accelerate the convergence speed of improved 2D LeNet-5 network.

### 5.4. Analysis of Generalization Ability of Fault Diagnosis Models

In this subsection, an experiment is conducted to verify the generalization ability of the proposed rolling-element bearing fault diagnosis models. For the improved 2D LeNet-5 network, the dataset composed of gray images is divided into training set and test set according to different ratios including 9:1, 8:2, 7:3, 6:4, 5:5, 4:6, 3:7, 2:8 and 1:9. For the improved 1D LeNet-5 network, the dataset composed of 1D raw vibration signals is also divided into training set and test set according to nine different ratios. In this experiment, the number of iterations is set to 150, and five times model training and test are performed according to different ratios of training set to test set. Table 8 presents the average accuracy of rolling-element bearing fault diagnosis obtained under different ratios of training set to test set.

As seen in Table 8, for the improved 2D LeNet-5 network, when the ratio of training set to test set is greater than or equal to 3:7, the average diagnosis accuracy can reach over 90%; for the improved 1D LeNet-5 network, when the ratio of training set to test set is greater than or equal to 4:6, the average diagnosis accuracy also can reach over 90%. The results show that the proposed fault diagnosis models have good generalization, this is mainly because the dropout operation and BN are introduced into improved 2D LeNet-5 network and improved 1D LeNet-5 network to enhance the generalization ability of the model. However, when the ratio of training set to test set is less than or equal to 2:8, the fault diagnosis accuracy of improved 2D LeNet-5 network and improved 1D LeNet-5 network are not satisfactory, this is because the training samples are too little, causing the problem of over-fitting, namely the model has good performance in the training set but poor performance in the test set.

It can be noted from Table 8 that the improved 2D LeNet-5 network achieves a higher fault diagnosis accuracy compared with the improved 1D LeNet-5 network when the ratio of training set to test set is less than or equal to 5:5. The results show that the generalization ability of improved 2D LeNet-5 network is stronger that that of improved 1D LeNet-5 network.

### 5.5. Comparison with Other Fault Diagnosis Methods

To further verify the effectiveness of the proposed rolling-element bearing fault diagnosis methods, the performance of improved 2D LeNet-5 network and improved 1D LeNet-5 network are compared with that of the other nine different fault diagnosis methods based on machine learning or deep learning, including SVM [8], k-NN [9], K-Means [10], BPNN [11], compact 1D CNN without fine-tuning [27], AlexNet [23], VGG-19 [24], ResNet-50 [25] and traditional LeNet-5 network [31].

For SVM, k-NN, K-Means and BPNN, the preprocessing of experimental data provided by CWRU is as follows: firstly every 4096 pieces of continuous raw vibration signals are divided into a sample, then each sample is decomposed by three-layer wavelet packet [40] to construct the eigenvectors of different conditions of rolling-element bearing, and finally the dataset composed of eigenvectors is divided into training set and test set according to the ratio of 7:3. For the compact 1D CNN and improved 1D LeNet-5 network, they take 1D raw vibration signals as input and use the same dataset presented in Table 4. For AlexNet, VGG-19, ResNet-50, traditional LeNet-5 network and improved 2D LeNet-5 network, they take 2D gray images of 64×64 pixels as input and use the same dataset presented in Table 2. In addition, the output layer of compact 1D CNN, AlexNet, VGG-19, ResNet-50 and traditional LeNet-5 network uses four neurons to classify the input data into normal condition, inner-race fault, ball fault and outer-race fault.

The settings of the most important parameters of different fault diagnosis methods based on machine learning are as follows.

- SVM: the penalty parameter *C* is set to 1, the radial basis function is chosen as the kernel function and the parameter γ is set to 0.125.
- k-NN: the number of nearest neighbors is set to 5.
- K-Means: the maximum number of iterations is set to 1000 and the number of clusters is set to 4.
- BPNN: the number of input layer nodes is set to 8, the number of hidden layer nodes is set to 12, the number of output layer nodes is set to 4, the learning rate is set to 0.003 and the maximum number of iterations is set to 1000.

The settings of network structure and hyper-parameters of compact 1D CNN, AlexNet, VGG-19 and ResNet-50 can be found in [27,41,42,43], respectively. The setting of network structure of traditional LeNet-5 network is shown in Table 1, and the setting of hyper-parameters of traditional LeNet-5 network is the same with that of improved 2D LeNet-5 network, which is shown in Table 6.

**Figure 13:**
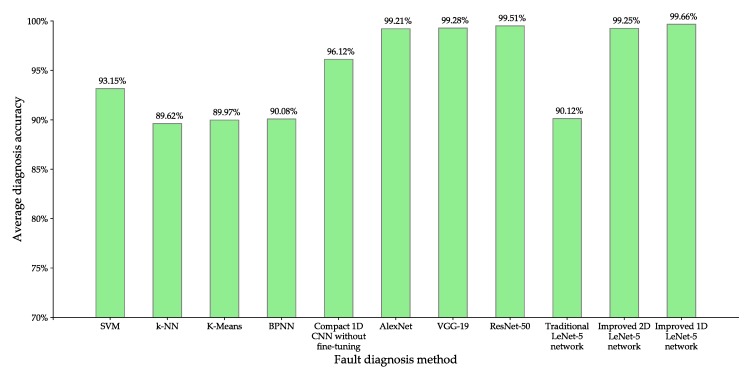
Accuracy comparison of different fault diagnosis methods.

In this experiment, for each fault diagnosis method, after the satisfactory fault diagnosis model is obtained through many training, the model is tested 100 times and the average diagnosis accuracy is obtained. Figure 13 presents the accuracy comparison of eleven different fault diagnosis methods based on machine learning or deep learning, and Table 9 presents the training time and model size comparison of seven different fault diagnosis methods based on deep learning.

From Figure 13, it can be seen that the proposed improved 2D LeNet-5 network achieves 6.10% diagnosis accuracy improvement over SVM, 9.63% over k-NN, 9.28% over K-Means and 9.17% over BPNN. The results show that the performance of these fault diagnosis methods based on machine learning is inferior to that of the proposed improved 2D LeNet-5 network, the main reasons are summarized as follows: for SVM, it is difficult to fine-tune the parameters *C* and γ especially for the complicated multi-class classification problem such as rolling-element bearing fault classification; for k-NN, it performs poorly on small training samples, while the normal condition and inner-race fault with fault diameter of 0.014 have relatively few samples on the CWRU dataset; for K-Means, it randomly selects the initial clustering centers, resulting in instable clustering effect; for BPNN, its weights are easy to converge to local minimum, causing it to fall into local optimal solution.

As shown in Figure 13 and Table 9, the difference of diagnosis accuracy of AlexNet, VGG-19, ResNet-50 and the proposed improved 2D LeNet-5 network is very small, but the training time of AlexNet, VGG-19 and ResNet-50 are 1.67 times, 2.39 times and 4.24 times as long as that of improved 2D LeNet-5 network respectively. This is mainly because that the network structures of AlexNet, VGG-19 and ResNet-50 are more complicated than that of improved 2D LeNet-5 network, for example, ResNet-50 has 49 convolution layers, one full-connection layer and 25.5 million parameters. It can be noted from Table 9 that the model sizes of AlexNet, VGG-19 and ResNet-50 are much greater than that of improved 2D LeNet-5 network.

Compared with traditional LeNet-5 network, the average diagnosis accuracy of improved 2D LeNet-5 network and improved 1D LeNet-5 network are increased by 9.13% and 9.54% respectively, and the training time of improved 2D LeNet-5 network and improved 1D LeNet-5 network are decreased by 30.03% and 55.53% respectively. The results show that the proposed improved 2D LeNet-5 network and improved 1D LeNet-5 network achieve a significant performance improvement than traditional LeNet-5 network.

It can be noted from Figure 13 and Table 9 that the average diagnosis accuracy of compact 1D CNN without fine-tuning is 3.13% lower than that of improved 2D LeNet-5 network, the average diagnosis accuracy of improved 1D LeNet-5 network is slightly higher than that of improved 2D LeNet-5 network, but the training time of compact 1D CNN and improved 1D LeNet-5 network are decreased by 58.07% and 36.44% than that of improved 2D LeNet-5 network respectively. The results show that compact 1D CNN and improved 1D LeNet-5 network can get competitive fault diagnosis accuracy with less training time. Compared with improved 2D LeNet-5 network, the improved 1D LeNet-5 network has the following advantages: (i) it does not require any preprocessing for the input data, this is because it directly takes 1D raw vibration signals as input; (ii) it has a faster training speed, this is because the 1D vector operations performed by 1D CNN have a lower computational complexity than the 2D matrix operations performed by 2D CNN. In terms of processing speed and practicality, currently the best way for classifying 1D vibration signals of rolling-element bearing is to use 1D CNN.

### 5.6. Performance Comparison under Noise Environment

**Figure 14:**
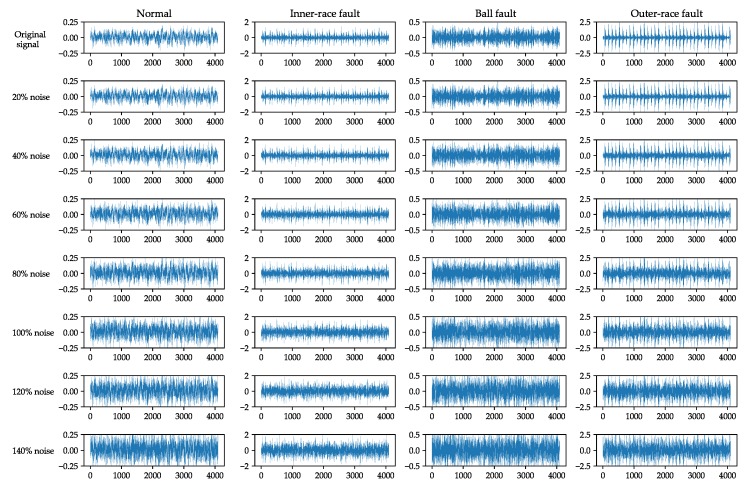
Examples of waveforms of rolling-element bearing vibration signals with different percentages of added noise.

In practical industrial production, the rolling-element bearing often continuously works in harsh environment, the collected vibration signals may contain a lot of noise, which would seriously affect the accuracy of rolling-element bearing fault diagnosis. In this subsection, an experiment is carried out to compare the fault diagnosis accuracy of the proposed improved 1D LeNet-5 network with that of the proposed improved 2D LeNet-5 network under noise environment. In this experiment, firstly the additive white Gaussian noise is added to the original vibration signals provided by CWRU according to seven different percentages of added noise including 20%, 40%, 60%, 80%, 100%, 120% and 140%. Figure 14 illustrates some examples of waveforms of rolling-element bearing vibration signals with different percentages of added noise. Secondly, the vibration signals with different percentages of added noise are respectively divided into training samples and test samples according to the ratio of 7:3, and each sample contains 4096 pieces of continuous vibration data. Finally, the training and testing of the improved 1D LeNet-5 network and improved 2D LeNet-5 network are carried out based on these training samples and test samples. Note that each sample needs to be transformed into a gray image of 64×64 pixels before the training and testing of improved 2D LeNet-5 network.

**Figure 15:**
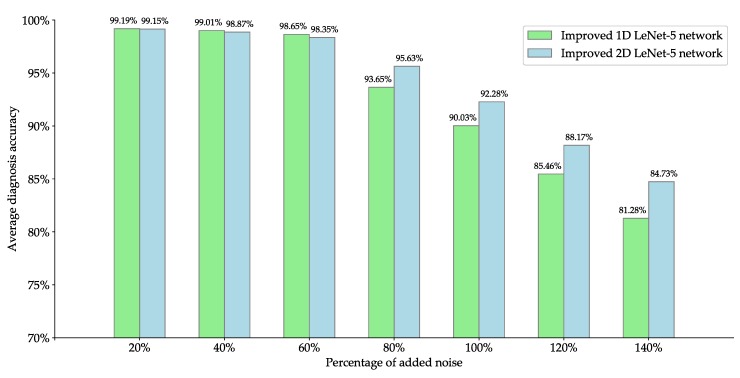
Comparison of fault diagnosis accuracy of improved 1D LeNet-5 network and improved 2D LeNet-5 network under noise environment.

Figure 15 presents the comparison of fault diagnosis accuracy of improved 1D LeNet-5 network and improved 2D LeNet-5 network under noise environment. As shown in Figure 15, when the percentage of added noise is less than or equal to 60%, the fault diagnosis accuracy of improved 1D LeNet-5 network and improved 2D LeNet-5 network can reach over 98%, and the improved 1D LeNet-5 network performs slightly better than improved 2D LeNet-5 network. With the increase of noise, the fault diagnosis accuracy decreases gradually. When the percentage of added noise varies from 80% to 140%, the fault diagnosis accuracy of improved 1D LeNet-5 network decreases from 93.65% to 81.28%, and the fault diagnosis accuracy of improved 2D LeNet-5 network decreases from 95.63% to 84.73%. The results show that the improved 1D LeNet-5 network and improved 2D LeNet-5 network have a certain anti-noise ability. It can be noted from Figure 15 that the improved 2D LeNet-5 network obtain a higher fault diagnosis accuracy than improved 1D LeNet-5 network under strong noise environment, the results show that the anti-noise ability of improved 2D LeNet-5 network is stronger than that of improved 1D LeNet-5 network.

## 6. Conclusions

In this paper, two different rolling-element bearing fault diagnosis methods are proposed, and they are verified by the rolling-element bearing data provided by CWRU. For the proposed fault diagnosis method based on the improved 2D LeNet-5 network, firstly the 1D raw vibration signals are transformed into 2D gray images and the histogram equalization is carried out on them to provide better input data for improved 2D LeNet-5 network, and then it performs 2D convolution and pooling operations on gray images through three convolution layers and three pooling layers. For the proposed fault diagnosis method based on the improved 1D LeNet-5 network, it directly takes 1D raw vibration signals as input without any preprocessing and performs 1D convolution and pooling operations on raw vibration signals through five convolution layers and five pooling layers. For the improved 2D LeNet-5 network and improved 1D LeNet-5 network, the BN and ReLU activation function are adopted after each convolution layer and the dropout operation is performed after each full-connection layer except the last layer, which can improve convergence speed and generalization ability. A series of experiments are conducted and the results prove the effectiveness of the proposed rolling-element bearing fault diagnosis methods. The results also show that the improved 1D LeNet-5 network performs better than improved 2D LeNet-5 network in most cases, and the improved 2D LeNet-5 network achieves a higher fault diagnosis accuracy than improved 1D LeNet-5 network under small training samples and strong noise environment.

In an actual production environment, the collected vibration data of rolling-element bearing is increasing and is more complicated, therefore the parallelization and optimization of the rolling-element bearing fault diagnosis method based on CNN and spark platform will be discussed in the future work.

## Figures and Tables

**Table 1:** Detailed settings of traditional LeNet-5 network structure.

Network Layer	Specific Settings	Number of Training Parameters	Output Characteristic
Input layer	a black-and-white image of 32×32 pixels	0	32×32×1
Conv1 layer	6 convolution kernels of size 5×5, stride = 1	156	28×28×6
Pool1 layer	pool size is 2×2, stride = 2	12	14×14×6
Conv2 layer	16 convolution kernels of size 5×5, stride = 1	1516	10×10×16
Pool2 layer	pool size is 2×2, stride = 2	32	5×5×16
FC1 layer	120 neurons	48120	1×1×120
FC2 layer	84 neurons	10164	1×84
FC3 layer	10 neurons	840	1×10

**Table 2:** Division of dataset composed of gray images.

Sample Type	Number of Training Samples					Number of Test Samples			
	0 HP	1 HP	2 HP	3 HP		0 HP	1 HP	2 HP	3 HP
N	126	252	252	252		54	108	108	108
I007	189	315	315	315		81	135	135	135
I014	105	252	315	315		45	108	135	135
I021	189	315	315	315		81	135	135	135
B007	189	315	315	315		81	135	135	135
B014	189	315	315	315		81	135	135	135
B021	189	315	315	315		81	135	135	135
O007	189	315	315	315		81	135	135	135
O014	189	315	315	315		81	135	135	135
O021	189	315	315	315		81	135	135	135

**Table 3:** Detailed settings of improved 2D LeNet-5 network structure.

Network Layer	Specific Settings	Number of Training Parameters	Output Characteristic
Input layer	a gray image of 64×64 pixels	0	64×64×1
Conv1 layer	8 convolution kernels of size 8×8, stride = 1	520	57×57×8
Pool1 layer	pool size is 2×2, stride = 2	16	28×28×8
Conv2 layer	32 convolution kernels of size 8×8, stride = 1	8672	21×21×32
Pool2 layer	pool size is 2×2, stride = 2	64	10×10×32
Conv3 layer	64 convolution kernels of size 5×5, stride = 1	57,920	6×6×64
Pool3 layer	pool size is 2×2, stride = 2	128	3×3×64
FC1 layer	120 neurons	69,240	1×1×120
FC2 layer	84 neurons	10,164	1×84
FC3 layer	4 neurons	336	1×4

**Table 4:** Division of dataset composed of 1D raw vibration signals.

Fault Type	Fault Diameter (inch)	Number of Training Samples					Number of Test Samples			
		0 HP	1 HP	2 HP	3 HP		0 HP	1 HP	2 HP	3 HP
Normal	0	126	252	252	252		54	108	108	108
Inner-race	0.007	189	315	315	315		81	135	135	135
Inner-race	0.014	105	252	315	315		45	108	135	135
Inner-race	0.021	189	315	315	315		81	135	135	135
Ball	0.007	189	315	315	315		81	135	135	135
Ball	0.014	189	315	315	315		81	135	135	135
Ball	0.021	189	315	315	315		81	135	135	135
Outer-race	0.007	189	315	315	315		81	135	135	135
Outer-race	0.014	189	315	315	315		81	135	135	135
Outer-race	0.021	189	315	315	315		81	135	135	135

**Table 5:** Detailed settings of improved 1D LeNet-5 network structure.

Network Layer	Specific Settings	Number of Training Parameters	Output Characteristic
Input layer	a sample composed of 4096 pieces of vibration data	0	4096×1
Conv1 layer	6 convolution kernels of size 64×1, stride = 1	390	4033×6
Pool1 layer	pool size is 8×1, stride = 8	48	504×6
Conv2 layer	16 convolution kernels of size 64×1, stride = 1	3856	441×16
Pool2 layer	pool size is 4×1, stride = 4	64	110×16
Conv3 layer	16 convolution kernels of size 16×1, stride = 1	1392	95×16
Pool3 layer	pool size is 2×1, stride = 2	32	47×16
Conv4 layer	32 convolution kernels of size 8×1, stride = 1	1344	40×32
Pool4 layer	pool size is 2×1, stride = 2	64	20×32
Conv5 layer	32 convolution kernels of size 4×1, stride = 1	1112	17×32
Pool5 layer	pool size is 2×1, stride = 2	64	8×32
FC1 layer	120 neurons	30,840	1×120
FC2 layer	84 neurons	10,164	1×84
FC3 layer	4 neurons	336	1×4

**Table 6:** Parameters settings of improved 2D LeNet-5 network and improved 1D LeNet-5 network.

Parameter Name	Parameter Value
Initial learning rate	0.008
Momentum	0.9
Gradient descent step-size	0.5
Training batch size	128
Test batch size	128

**Table 7:** Fault diagnosis accuracy of different conditions of rolling-element bearing.

Fault Diagnosis Model	Fault Diagnosis Accuracy (%)										
	N	I007	I014	I021	B007	B014	B021	O007	O014	O021	Average
Improved 2D LeNet-5 network	99.73	99.58	98.81	99.17	98.55	98.97	99.79	99.17	99.58	99.17	99.25
Improved 1D LeNet-5 network	99.78	99.68	99.81	99.49	99.60	99.43	99.62	99.89	99.81	99.50	99.66

**Table 8:** Average Diagnosis accuracy obtained under different ratios of training set to test set.

Fault Diagnosis Model	Average Diagnosis Accuracy								
	9:1	8:2	7:3	6:4	5:5	4:6	3:7	2:8	1:9
Improved 2D LeNet-5 network	99.36%	98.80%	98.66%	96.04%	94.89%	93.39%	90.42%	81.77%	69.12%
Improved 1D LeNet-5 network	99.71%	99.35%	99.11%	97.38%	93.64%	90.74%	85.59%	72.80%	58.59%

**Table 9:** Training time and model size comparison of different fault diagnosis methods.

Fault Diagnosis Method	Training Time (s)	Model Size (MB)
Compact 1D CNN without fine-tuning	252	0.7
AlexNet	1006	77.4
VGG-19	1434	188
ResNet-50	2546	90.2
Traditional LeNet-5 network	859	1.3
The proposed improved 2D LeNet-5 network	601	0.6
The proposed improved 1D LeNet-5 network	382	1.2
